# Opportunities for Tuberculosis Diagnosis and Prevention among Persons Living with HIV: A Cross-Sectional Study of Policies and Practices at Four Large Ryan White Program-Funded HIV Clinics

**DOI:** 10.1371/journal.pone.0101313

**Published:** 2014-07-07

**Authors:** Lisa Pascopella, Julie Franks, Suzanne M. Marks, Katya Salcedo, Kjersti Schmitz, Paul W. Colson, Yael Hirsch-Moverman, Jennifer Flood, Jennifer Sayles

**Affiliations:** 1 Tuberculosis Control Branch, Division of Communicable Disease Control, Center for Infectious Diseases, California Department of Public Health, Richmond, California, United States of America; 2 The International Center for AIDS Care and Treatment Programs (ICAP)/Columbia University Mailman School of Public Health, New York, New York, United States of America; 3 Division of Tuberculosis Elimination, Centers for Disease Control and Prevention, Atlanta, Georgia, United States of America; 4 Office of AIDS Programs and Policy, Los Angeles County Department of Public Health, Los Angeles, California, United States of America; The University of Hong Kong, Hong Kong

## Abstract

**Objective:**

We describe the frequency and attributes of tuberculosis testing and treatment at four publicly-funded HIV clinics.

**Methods:**

We abstracted medical records from a random sample of 600 HIV-infected patients having at least one clinic visit in 2009 at four clinics in New York and Los Angeles Metropolitan Statistical areas. We described testing and treatment for tuberculosis infection (TBI), 2008–2010, and estimated adjusted odds ratios (aORs). We interviewed key informants and described clinic policies and practices.

**Results:**

Of 600 patients, 500 were eligible for testing, and 393 (79%) were tested 2008–2010; 107 (21%) did not receive at least one tuberculin skin test or interferon gamma release assay. Results were positive in 20 (5%) patients, negative in 357 (91%), and unknown in 16 (4%). Fourteen (70%) of 20 patients with TBI initiated treatment at the clinics; only three were documented to have completed treatment. Three hundred twenty three (54%) patients had chest radiography, 346 (58%) had tuberculosis symptom screening, and three had tuberculosis disease (117 per 100,000 person-years, 95% confidence interval (CI) = 101–165). Adjusting for site, non-Hispanic ethnicity (aOR = 4.9, 95% CI = 2.6–9.5), and employment (aOR = 1.9, 95% CI = 1.0–3.4) were associated with TBI testing; female gender (aOR = 2.0, 95% CI = 1.4–3.3), non-black race (aOR = 1.7, 95% CI = 1.3–2.5), and unemployment (aOR = 1.5, 95% CI = 1.1–2.1) were associated with chest radiography. Clinics evaluated TBI testing performance annually and identified challenges to TB prevention.

**Conclusions:**

Study clinics routinely tested patients for TBI, but did not always document treatment. In a population with a high TB rate, ensuring treatment of TBI may enhance TB prevention.

## Introduction

Persons living with HIV (PLWH) have a 20 to 37-fold increased risk of active tuberculosis (TB) disease compared with persons who are not infected with HIV[1]. Even in settings with low TB incidence, low HIV prevalence, and access to highly active antiretroviral therapy (HAART), PLWH are at high risk for TB, with an estimated incidence of 215 per 100,000 in the first three months of HAART [2]. Independent risk factors for TB after HAART initiation in the United States (U.S.) and Canada include baseline CD4 lymphocyte count less than 200, non-white race, Hispanic ethnicity, and history of injection drug use [2]. To prevent TB among PLWH, U.S. guidelines recommend prompt identification and treatment of TB infection (TBI). TBI testing with the tuberculin skin test (TST) or an interferon gamma release assay (IGRA) on whole blood is recommended at entry into HIV care and annually for those at substantial risk of exposure to *Mycobacterium tuberculosis* [3]. A positive result should trigger rapid evaluation for active TB disease, including screening for TB symptoms and chest radiography [3]. HIV-infected individuals who test positive for TBI without evidence of TB disease should receive treatment for TBI [3]. Additionally, any patient with newly diagnosed HIV should be evaluated for TB disease with assessment of patient history and chest radiography [4].

Past studies have shown that these guidelines are not followed among a substantial number of PLWH; only one-half to two-thirds of patients in care for HIV underwent TBI testing and only two-thirds of newly identified TBI patients received treatment [5]–[7]. We hypothesized that TB services for PLWH had improved since these publications, so we sought to determine the frequency and attributes of TB detection and prevention activities at four publicly-funded HIV clinics in 2008–2010, at a time when TB disease and TB-HIV co-infection had declined [8].

## Methods

### Ethics Statement

The U.S. Centers for Disease Control and Prevention Institutional Review Board B and the California Health and Human Services Agency's Committee for the Protection of Human Subjects (CPHS) and the Los Angeles County Public Health Institutional Review Board and each of four clinics Institutional Review Boards (IRBs) approved the study and granted a waiver of patient informed consent and patient authorization for the clinic record reviews. (All three criteria for such a waiver were satisfied: 1) the use or disclosure of protected health information (PHI) involves no more than a minimal risk to the privacy of individuals, 2) the research could not practicably be conducted without the waiver, and 3) the research could not be conducted without access to and use of the PHI.) All institutional review boards that approved the study also approved the following approach. All participating clinic staff interviewed gave written informed consent. However, to eliminate the possibility of identification of the interviewee through linkage of consent form signatures, a waiver of documentation of informed consent under 45 CFR 46.117(c) enabled the use of a check box rather than a signature to indicate consent was obtained; the interviewer then signed the consent form.

### Study population

Two U.S. Metropolitan Statistical Areas (MSA) with elevated HIV and TB prevalence were selected: New York City and Los Angeles. The Ryan White HIV/AIDS Program (RWP) of the Health Resources and Services Administration (HRSA) addresses the health needs of low-income PLWH by funding primary HIV care (http://hab.hrsa.gov). Two RWP Part C-funded HIV clinics from each MSA having at least 450 patients annually participated. All four clinics served predominantly racial/ethnic minority populations: two were hospital-based, one was a private community clinic, and one was a health department clinic. All clinics participated in the HRSA quality assurance program [9].

### Data collection and analysis

Study staff abstracted medical records of a random sample of 600 HIV-infected patients having at least one clinic visit in 2009 (150 consecutive patients from each clinic). We examined patient characteristics and TB services outcomes (i.e., frequency having TBI tests, TBI treatment, TB symptom screening, chest radiography, and TB disease) during 2008–2010. We concluded data collection on December 31, 2010. We assumed that chest radiographs were used for TB evaluation. We used multivariable logistic regression, with a backwards selection strategy, to identify patient characteristics that were independently associated with TBI testing and chest radiography. Variables assessed in the TBI testing and chest radiography models included those associated with characteristics in Table 1. We applied a threshold p-value of 0.05 to keep the variable in the model.

**Table 1:** Patient population characteristics.

Characteristic	N (%)
Clinic site	
Los Angeles MSA	300 (50)
New York MSA	300 (50)
Demographic	
Gender	
Male	419 (70)
Female	175 (29)
Transgender	6 (1)
Sexual Identity	
Straight/heterosexual	197 (33)
Gay/Men who have sex with men/Lesbian/women who have sex with women	159 (27)
Bisexual	47 (8)
Other	6 (1)
Unknown	191 (32)
Age, median (range)	46 (18–86)
18-24	12 (2)
25-44	254 (42)
45-64	307 (51)
65+	27 (5)
Race/ethnicity	
Hispanic	246 (41)
White	20 (3)
Black	281 (47)
American Indian/Alaskan Native	5 (1)
Asian	4 (1)
Native Hawaiian/other Pacific Island	1 (<1)
Other	4 (1)
Unknown	39 (7)
Nativity	
U.S.-born	258 (43)
Not U.S.-born	157 (26)
Unknown	185 (31)
Country of birth a	
Mexico	97 (62)
El Salvador	13 (8)
Dominican Republic	7 (4)
Guatemala	6 (4)
Cuba	5 (3)
Other Central/South American nation	19 (12)
African nation	6 (4)
Asian nation	3 (2)
Middle Eastern nation	1 (1)
Language	
English native	246 (41)
Spanish native	191 (32)
Other language native	5 (1)
Unknown	158 (26)
Social/behavioral/economic	
Injection drug use within past year	
Yes	57 (10)
No	388 (65)
Unknown	155 (26)
Non-injection drug use within past year	
Yes	149 (25)
No	268 (45)
Unknown	183 (31)
Excess alcohol use within past year	
Yes	63 (11)
No	368 (61)
Unknown	169 (28)
Homeless within past year	
Yes	58 (10)
No	354 (59)
Unknown	188 (31)
Incarceration within past year	
Yes	71 (12)
No	119 (20)
Unknown	410 (68)
Clinical	
Years at clinic, median (range)	7 (1–21)
1–2.9	143 (24)
3–4.9	79 (13)
5–9	193 (32)
10 or more	184 (31)
Unknown	1 (<1)
Years HIV positive b, median (range)	8.6 (1–28)
CD4 nadir level at any time, median (range)	182 (1–1022)
0–199	317 (53)
200–349	158 (26)
350–499	68 (11)
500 or over	52 (9)
Unknown	5 (1)
CD4 level closest to TBI test c, median (range)	437 (2–1458)
0–199	87 (15)
200–349	95 (16)
350–499	100 (17)
500 or over	196 (33)
Unknown	122 (20)
Viral load closest to TBI test c, median (range)	50 (0–5,410,000)
Zero	204 (34)
1–9,999	192 (32)
10,000–49,999	40 (7)
50,000–99,999	12 (2)
100,000 or over	26 (4)
Unknown	126 (21)
Ever on HAART	
Yes	573 (96)
No	25 (4)
Unknown	2 (<1)
On HAART at initial clinic visit	
Yes	200 (33)
No	250 (42)
Unknown	150 (25)
Number of HAART starts while at clinic, median (range)	1 (1–9)
1	418 (70)
2	64 (11)
3	37 (6)
4	16 (3)
5 and more	14 (2)
Unknown	51 (9)
Co-morbidities	
Diabetes mellitus	68 (11)
Cancer	21 (4)
Prolonged corticosteroids	18 (3)
Hematologic disease	5 (1)
End-stage renal disease	5 (1)
Contact to infectious TB case	2 (<1)
Had TBI/TB prior to 2008	
Yes prior TBI	79 (13)
Yes prior TB disease	21 (4)
No	500 (83)

a Of those born outside U.S.

b Before December 31, 2010.

c Within 6 months before or after TST/QFT.

We weighted each observation of patient-level data by the probability of selection at the clinic (inverse of the total clinic population divided by 150). We estimated the annual rates of TBI testing, and chest radiography by dividing the total number of tests by the number of person-years at the clinic. To estimate the TB disease rate, we first calculated the weighted rate per year; then selected the median rate and estimated the non-parametric 95% confidence interval. SAS version 9.2 (SAS Institute Inc., Cary, North Carolina) was used for statistical analyses.

We collected and abstracted written clinic policies, relevant to TBI and TB testing, diagnosis, and treatment; and, observed clinic environment and encounters with patients. Clinic directors (N = 4) and TB program directors (N = 3) identified their staff who had sufficient program knowledge to be key informants for the study. Trained study staff conducted face-to-face key informant interviews using a semi-structured instrument, and verified responses by audio recordings. We reviewed and used data from written policies, clinic observations, and key informant interviews with clinic and TB program staff to describe practices of TBI testing, TBI treatment, TB disease evaluation, and linkages with TB programs.

## Results

### Patient characteristics, TB services outcomes, and TB disease rate

We present characteristics of the 600 patients in Table 1. Two-thirds of study patients had been in care for HIV at their clinic for at least five years. The majority of patients were male (N = 419, 70%), and of black race (N = 281, 47%) or Hispanic ethnicity (N = 246, 41%). Of those with known birth country (N = 415), 157 (38%) were foreign-born, with 97 (62%) born in Mexico. Many patients had risk factors for TB, including history of: non-injection drug use (N = 149, 25%), excess alcohol consumption (N = 63, 11%), injection drug use (N = 57, 10%), incarceration (N = 71, 12%), and homelessness (N = 58, 10%). Only two patients had documented contact with a person with infectious TB. In addition to HIV infection, patients had other risk factors for TB disease: 68 (11%) had diabetes; 21 (4%) had cancer, 18 (3%) were on prolonged corticosteroid therapy, five (1%) had hematologic disease, and five (1%) had end-stage renal disease.

The majority (N = 317, 53%) of patients had a nadir CD4 count of less than 200. While nearly all (N = 573, 96%) had been on HAART at some time during the study period, 22% were known to have started HAART more than once while at the clinic. Patients without history of TBI or TB were eligible for annual TB testing, per study clinic policy (see below). Thus, 500 patients (83%) were eligible for TBI tests in 2008–2010 because they did not have a history of TBI or TB prior to 2008 (Table 1). One hundred patients (17%) had TBI (N = 79) or TB disease (N = 21) prior to 2008.

**Figure 1:**
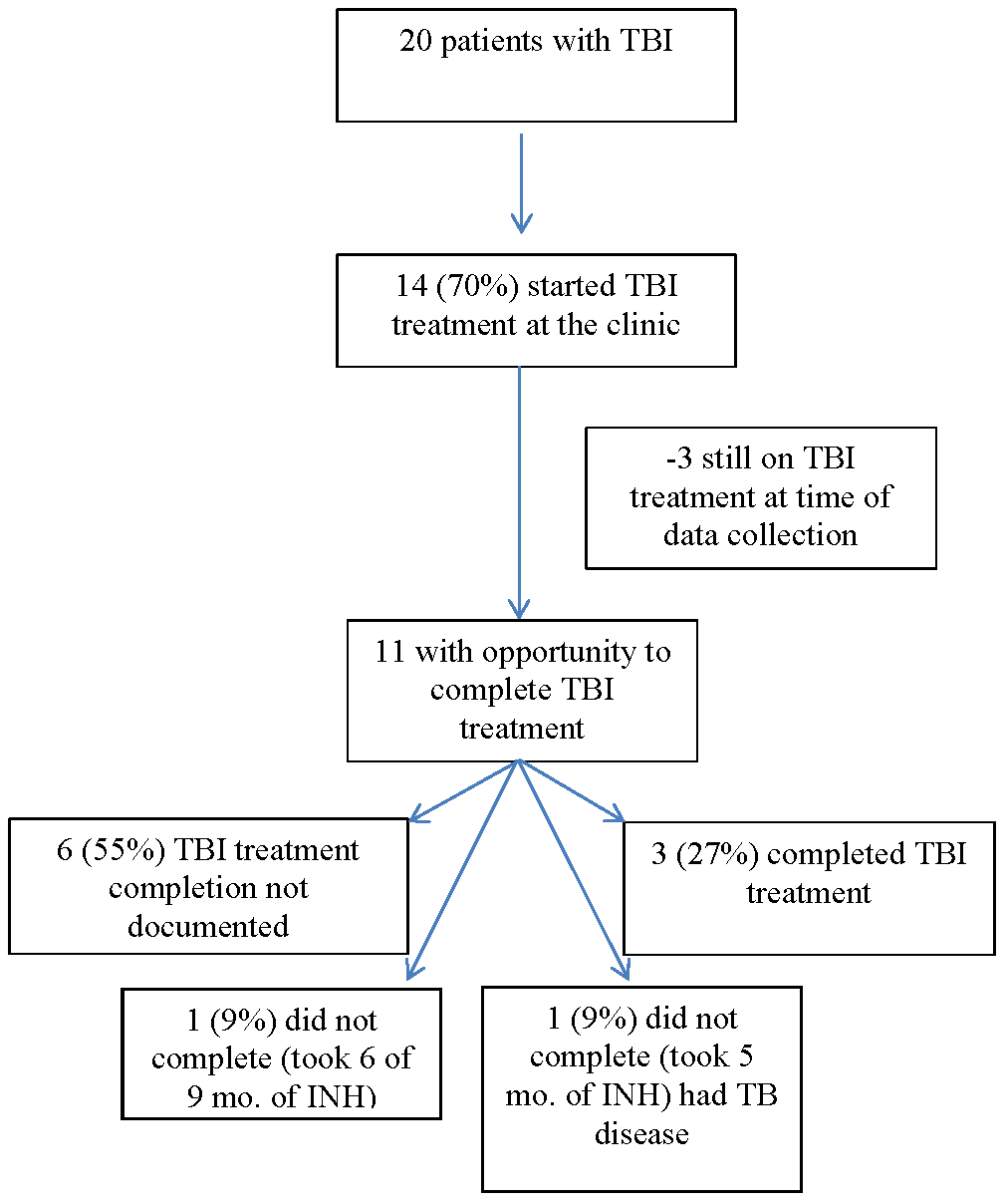
Frequency and percent of TBI patients receiving and completing TBI treatment.

As shown in Table 2, 393 (79%) of 500 eligible patients were tested with either a TST or IGRA at least once during 2008–2010. Of 393 tested, 20 (5%) patients had positive, 357 (91%) had negative, 14 (4%) had no documented TST results, and two had indeterminate IGRA results. Of the 20 newly diagnosed TST/IGRA-positive patients, 13 (65%) had documented TB symptom screening, and 19 (95%) had documented chest radiography during 2008–2010. Of the 20 patients who tested positive for TBI, 14 (70%) initiated TBI treatment at the clinic (see Figure 1). Three (15%) patients were still on treatment when staff concluded data collection. Excluding the three patients who were still on treatment, only three (27%) of 11 completed treatment (i.e., medical record documented 9 months of INH); two (18%) did not complete; and six (55%) had no documentation of treatment completion. One patient who did not complete treatment was diagnosed with and treated for TB disease (Figure 1).

**Table 2:** Frequency and percent of patients who received TB services, 2008–2010.

Patient history of TBI or TB disease prior to 2008	TST/IGRA N (%)	TB symptom-screening N (%)	Chest radiography N (%)
500 patients with no prior TBI/TB^a^	393 (79)	285 (57)	247 (49)
393 were tested with TST/IGRA		223 (57)	190 (48)
357 had negative TST/IGRA		199 (56)	160 (45)
20 had newly positive TST/IGRA		13 (65)	19 (95)
16 had unknown TST/IGRA result		11 (69)	11 (69)
107 were not tested with TST/IGRA		62 (58)	57 (53)
100 patients with prior TBI/TB^a^	17 (17)	61 (61)	76 (76)
79 with prior TBI	10 (13)	44 (56)	67 (85)
21 with prior TB	7 (33)	17 (81)	9 (43)

a Prior TBI/TB = documented with TBI or TB disease earlier than January 1, 2008.

Of the 357 TST/IGRA-negative patients, 199 (56%) had documented TB symptom screening, and 160 (45%) had documented chest radiography (Table 2). One TST-negative patient was fully evaluated and diagnosed with TB disease. Of the 107 patients not tested for TBI, 62 (58%) had documented TB symptom screening and 57 (53%) had documented chest radiography (Table 2).

Of the 79 patients who had TBI prior to 2008, 44 (56%) had documented TB symptom screening and 67 (85%) had documented chest radiography in 2008–2010. One patient in this group had completed treatment for TBI in 2000, but was diagnosed with TB disease in 2008, after chest radiography and specimen collection. Of the 21 patients with TB disease prior to 2008, 17 (81%) had documented TB symptom screening and 9 (43%) had documented chest radiography in 2008–2010 (Table 2).

Overall, among all 600 patients, 323 (54%) had documentation of chest radiography, and 346 (58%) had documentation of TB symptom screening at least once during 2008–2010. The median individual weighted rate of chest radiography was 0.7 times per year. For the 500 patients eligible for TBI testing, the median individual weighted rate of TST or IGRA testing was 0.7 times per year.

Three patients had TB disease during 2008–2010; the median weighted population rate of TB disease was 117 per 100,000 person years, with a 95% confidence interval of 101–165.

### Patient characteristics associated with TBI testing and chest radiography

Patient characteristics independently associated with TBI testing included: non-Hispanic ethnicity (adjusted odds ratio (aOR) = 4.9, 95% confidence interval (CI) = 2.6–9.5), receipt of care at a participating clinic in Los Angeles (aOR = 3.1, 95% CI = 1.5–6.4), and employment (aOR = 1.9, 95% CI = 1.0–3.4). Patient characteristics associated with having received a chest radiograph included: female gender (aOR = 2.0, 95% CI = 1.4–3.3); non-black race (aOR = 1.7, 95% CI = 1.3–2.5); and unemployment (aOR = 1.5, 95% CI = 1.1–2.1) (Table 3). Country of birth could not be assessed in the models due to a high frequency (31%) of missing data.

**Table 3:** Patient-level factors associated with TBI testing, and chest radiography.

Factors associated with TBI testing	Weighted adjusted OR (95% CI)
Non-Hispanic ethnicity	4.9 (2.6–9.5)
Received care at participating clinic in Los Angeles (ref = New York)	3.1 (1.5–6.4)
Employment	1.9 (1.0–3.4)
Factors associated with chest radiography	
Female gender	2.0 (1.4–3.3)
Non-black race	1.7 (1.3–2.5)
Unemployment	1.5 (1.1–2.1)

### Programmatic context of TB services

Key informants were interviewed in Los Angeles (n = 12, with 1 from the local TB program) and the New York MSA (n = 8, with 2 from local TB programs). Key informants from each of the four clinics reported that identification and prevention of TB was a high priority activity. However, written clinic policies did not specifically mention TBI testing and treatment, except for Clinic 4 which instructed staff to record annual dates and results of the tests (Table 4). Clinic 2 outlined the roles and responsibilities of clinic staff working with the local TB control program to provide directly observed therapy for patients with TB disease (Table 4). References for clinic policies were reported to be: local or state TB program manuals, national guidance on HIV primary care [10], and national guidelines [3], [11], [12].

**Table 4:** Clinic-specific rates, policies, procedures related to TB services.

Clinic No.	1	2	3	4
Clinic type	Health department	Private not-for-profit	Hospital	Public hospital
TBI testing^a^	0.9	0.7	0.4	1.0
Chest radiography^a^	0.6	0.4	0.7	1.0
Symptom screen/chest radiography documented? b	Chest radiography only	Chest radiography only	Symptom screen and chest radiography	Symptom screen and chest radiography
Written clinic policies referred to specific TBI testing or treatment procedures?	No; Described county health-department wide TBI and TB patient management and treatment procedures	No	No	Yes; Described clinic-specific procedures to record annual TST/IGRA dates and results
Written clinic policies referred to specific TB testing or treatment procedures?	No; Described county health-department wide TBI and TB patient management and treatment procedures	Yes; Described clinic-specific staff roles and responsibilities to coordinate the administration of directly observed therapy for TB patients with the local TB control program	No	Yes; Described clinic-TB program coordination
TB evaluation and follow- up procedures	Radiography in hospital across the street Patients with presumptive TB were referred to local TB clinic	Radiography in clinic Patients with presumptive TB were referred to local TB clinic. Clinic staff worked with local TB program staff to ensure directly observed therapy.	Radiography in building that houses clinic Followed facility infection control procedures for patients with presumptive TB and escorted to emergency department.	Radiography in building that houses clinic Patients with presumptive TB were referred to emergency department for hospital admission.
TBI treatment monitoring	Clinic-wide assessment Clinic nurses completed, and TB program nurses reviewed, individual patient tracking forms to identify gaps in testing and treatment.	Patient-specific assessment Staff called patients to remind them of follow-up visits.	Patient-specific assessment Staff called patients to remind them of follow-up visits.	Clinic-wide assessment Clinic staff directly communicated with TB program staff, and recorded patient treatment progress in the shared electronic medical record.
Clinic-TB program partnership activities	TB program provided consultation on TB and complex TBI cases at clinic. TB program nurses worked with clinic nurses to ensure complete data collection and follow-up for patients with TBI (no longer funded).	Clinic staff worked with TB program staff to ensure directly observed therapy of clients with active TB disease	TB program provided consultation and TB program staff member was assigned to follow up on facility patients receiving inpatient TB care or TBI treatment.	TB program provided care and follow-up of complex clinic patients with TBI. Shared institutional affiliation and shared electronic patient records facilitated exchange of information and consults. Infectious Diseases Specialist provided TB and HIV care at TB program co-located clinic and referred to HIV clinic for additional care (no longer funded)
Education for, and support of, patients undergoing TB and TBI services	Clinic nurses provided TB testing brochures to patients.	Clinic staff ensured continued HIV care of patients during at-home recovery from TB through phone calls and home visits. TB/HIV patient education brochures, in Spanish, were displayed.	Clinic staff utilized treatment adherence program, providing tools to patients (e.g., pill boxes), and counseling on treatment adherence.	TB and TB/HIV patient education brochures, in English and Spanish, were displayed.

a Median of individual annual weighted rate.

b Documented in patient medical records.

Informants from all clinics reported that TST placement and reading, or IGRA testing, and chest radiography, were provided during patients' initial evaluations, i.e., within three months of intake, and annually thereafter. Clinics 1 and 2 reported the use of TSTs only, whereas Clinics 3 and 4 used IGRAs in a small proportion of patients and TSTs in most patients. Clinics 1 and 2 planned to implement IGRAs in the coming year. Informants confirmed that patients with prior TBI or TB disease were to receive an annual chest radiograph to assess potentially active TB disease. All clinics referred patients with positive TSTs/IGRAs for chest radiography and physician evaluation to rule out TB disease prior to initiating TBI treatment. Although all clinics had similar methods to encourage return visits for TST reading, i.e., reminder phone calls and monitoring of return visits, and reviewed TBI test completion monthly or quarterly, only Clinics 1 and 4 partnered with their local TB program to perform systematic monitoring of TBI testing and treatment (Table 4).

Clinic patients with abnormal chest radiographs were referred to a clinic-associated hospital or to the local TB clinic for further assessment and TB treatment, if indicated (Table 4). Each clinic provided continuity of care for patients on anti-TB treatment. To support patients undergoing TB services, clinics provided medical interpreters for non-English-speaking patients, and three clinics displayed or provided TB-specific patient education materials (Table 4).

Although all clinics had access to consultation from their local health department TB program, the extent of the partnership with the TB program varied. Clinics 1 and 4 had the highest TBI test rates and reported the most frequent and routine interactions, including joint monitoring and assessment of TBI testing and treatment (Table 4). Clinic 1 partnered with its local TB program to perform systematic review of completion and outcomes of TBI testing and treatment and Clinic 4 shared and routinely reviewed patient data with its local TB program. Key informants from two of the three TB programs that interfaced with the clinics reported the recent loss of staff that performed partnership activities, e.g., monitoring of TBI testing and treatment of patients at HIV clinics. (Table 4).

### Perceived barriers and facilitators of TBI testing and treatment

In response to an interview question regarding barriers to TBI testing and treatment, key informants from three clinics reported that the follow-up visit required for reading the TST was a barrier for patient retention and completion of TBI testing. According to clinic staff, IGRAs, while desirable, were of limited utility because of their high cost and high frequency of indeterminate results. One key informant stated that barriers for completion of TBI treatment included the perception of a large pill burden, long (9-month) treatment regimen, and, for immigrant patients, language differences and uncertain legal status resulting in reluctance to present for care.

In response to an interview question regarding facilitators to TB testing and treatment, key informants noted the following: TB expertise among staff, having sufficient clinic supplies, use of IGRAs, positive relationships with local TB program staff and review of shared electronic medical records, use of electronic patient records and tracking systems to facilitate annual TBI testing, having staff trained in cultural competency, and the institutional recognition of the importance of TBI testing. Key informants from all clinics reported that TBI testing was an indicator in their HRSA quality management programs.

## Discussion

TBI testing was provided to a greater proportion of PLWH in our study (79%) than in previous studies, (52–65%) [5]–[7]. Possible reasons for improved TBI testing in our study compared to previous studies include underlying differences in the study populations (e.g. our study population included mostly long-term clients of four RWP Part C-funded clinics, whereas one of the previous studies included only patients with newly diagnosed HIV infection), increased awareness of TB screening recommendations through time (previous studies examined patients-in-care as early as 1995 and as recent as 2008), and/or greater performance at our study clinics which participated in HRSA's quality improvement program. Completion of an effective treatment regimen for TBI is a critical component of TB prevention and study clinics did not routinely document treatment outcomes.

While this study had small numbers, the occurrence of three TB cases in 600 patients suggests a high TB rate, which is consistent with published estimates of the TB rate in HIV-infected persons in California [8], and emphasizes the importance of screening for TB disease. Only two clinics documented TB symptom screening in patient medical records, limiting our ability to assess the extent of prompt TB detection activities. On the other hand, each clinic reported policies of annual chest radiographs for its patients; however, only 54% of patients had received a chest radiograph during a three year period.

Although Hispanic and black persons are at higher risk of TB than white persons [8], [13], Hispanic patients at the study clinics were less likely to have TBI testing, and black patients were less likely to receive chest radiography than other racial/ethnic groups, even after adjusting for other variables such as employment. We were unable to adjust for all potential confounding variables, such as insurance status and other factors associated with health services utilization so racial/ethnic disparities could not be further investigated.

Even though each clinic's goal was to provide annual TBI testing for all patients, one-fifth of the population did not receive at least one TST or IGRA during a three-year period. Each clinic used a variety of approaches to ensure annual TBI testing, including staff training, quality improvement programs, annual retrospective review of patient medical records, and real-time telephone contact to remind patients of and/or reschedule TST reading visits. Key informants from three clinics stated that a barrier to annual testing was the challenge of the return visit for TST reading, even though only 4% of patients had undocumented TST results. Although replacement of TSTs with IGRAs may overcome this barrier, many key informants were concerned about IGRA test performance in HIV-infected populations, and had a perception that IGRAs produced an unacceptably high frequency of indeterminate results. However, study patient data revealed that only 1% of patients tested with IGRAs had an indeterminate result. The two clinics that were performing only TSTs during the study period planned to implement IGRAs. IGRAs have greater specificity (i.e., fewer false-positives) than TST [14], and may identify fewer patients requiring TBI treatment. However, a subset of PLWH will have false-negative TST or IGRA test results, which will require monitoring for TB disease or TBI treatment [15].

One-third of the patients with TBI during 2008–2010 did not initiate TBI treatment at the clinic. If these patients were not treated elsewhere, the clinics missed an opportunity to prevent TB. If lack of documentation was equivalent to lack of treatment, then the majority of patients with TBI who initiated treatment in this study did not complete treatment for TBI, and therefore remained at high risk for progression to TB disease. Since antiretroviral therapy is associated with decreased incidence of TB [16], patients who were non-adherent to HAART (approximately one-fifth of patients started HAART multiple times) would be an important group for TB prevention. Another risk for progression from TBI to TB disease is diabetes mellitus (DM) [17], which affected a substantial percentage (11%) of the study patients. In fact, one of the study patients with TBI had DM and did not complete treatment and represents a crucial missed prevention opportunity. PLWH who also have DM are a group who would benefit from intensified TB prevention activities.

An important study finding was the absence of TBI treatment documentation. Each clinic used a HRSA quality assurance indicator to monitor testing for TBI but employed few, if any, performance evaluation methods to monitor TBI treatment. Additional indicators, such as the proportion of patients with TBI who started and completed treatment, should be implemented to improve documentation of treatment outcomes and TB prevention. Two of the study clinics had, in the past, worked with local health department TB program staff to measure progress towards treatment initiation and completion goals. The two clinics that performed assessment of TBI testing and treatment outcomes in partnership with their local TB programs were those that had the highest TBI testing rate. Perhaps private clinic-based TB prevention activities would improve with systematic review of testing and treatment performance indicators, which could be done in partnership with public health TB programs. A shortened TBI treatment regimen [18], if shown to be effective and safe in PLWH, may also improve treatment adherence and completion.

## Conclusions

In conclusion, the RWP HIV clinics in our study routinely tested patients for TBI, but did not always document treatment. In a population with a high TB rate, ensuring treatment of TBI may enhance TB prevention.
